# Rhein promotes skin wound healing by activating the PI3K/AKT signaling pathway

**DOI:** 10.1515/med-2024-1116

**Published:** 2024-12-19

**Authors:** Dong Yang, Wei Li, Ping Xiang, Tingrui Ge, Huazhuan Li, Yonggang Zhang

**Affiliations:** Department of Anorectal Surgery, The First People’s Hospital of Lianyungang, Lianyungang, 222016, China; Department of Hepatobiliary Surgery, The First People’s Hospital of Lianyungang, Lianyungang, 222016, China

**Keywords:** Rhein, skin wound healing, PI3K/AKT signaling pathway

## Abstract

Rhein is a natural anthraquinone substance extracted from *Rheum palmatum* L. This study aimed to evaluate Rhein’s protective effects against skin wound by *in vivo* and *in vitro* models and investigate whether its protective mechanism regulated the PI3K/AKT signaling pathway. The skin wound mice model was established and then treated with Rhein for 10 days. Hematoxylin and eosin staining and Masson’s trichrome staining were applied to assess histological changes and collagen maturity in the mice skin wound tissues. Human skin fibroblasts (HSFs) viability, migration, and invasion were detected by Cell counting kit-8 (CCK-8), scratch wound, and transwell assays respectively. Moreover, the protein expression of p-PI3K, PI3K, p-AKT, and AKT were determined by western blot assay. We found that local treatment with Rhein promoted skin wound healing and accelerated collagen maturation, compared with the Model group. In addition, Rhein promoted skin wound healing through accelerated HSF proliferation, migration, and invasion. Furthermore, Rhein remarkably enhanced p-PI3K and p-AKT expression, as well as p-PI3K/PI3K and p-AKT/AKT ratio in skin wound mice and HSF cells, suggesting that Rhein promoted skin wound healing by activating PI3K/AKT signaling pathway. In conclusion, Rhein is a promising agent for promoting wound healing of skin tissues.

## 1 Introduction

Wound healing is a complex process that refers to the repair process of tissue defects caused by external forces on the body [1,2]. The healing of skin wounds requires the coordination of multiple processes, including inflammatory response, cell migration and proliferation, collagen synthesis, and deposition [3,4]. There were different degrees of tissue necrosis and vascular rupture in the wound area during the inflammatory phase, followed by inflammatory reactions within a few hours [5]. In the proliferative phase, the basal cells at the wound edge began to proliferate and migrate to the center of the wound, forming a single layer of epithelium covering the surface of granulation tissue. When these cells meet, they stop migrating and proliferate and differentiate into squamous epithelium [6]. At present, increasing researches focus on finding methods for skin wound healing [5,7,8]. Although we have made significant progress in understanding how wounds heal in an orderly manner, the mechanisms controlling the behavior of repair cells remain incompletely understood.

As a traditional Chinese medicine, *Rheum palmatum* L. has a medicinal history of over a thousand years in China. At present, pharmacological studies of *R. palmatum* L. mainly focus on anthraquinones, including rhein, emodin, and chrysophanol [9,10]. The pharmacological effects of *R. palmatum* L. mainly include anti-tumor, anti-bacterial, anti-inflammatory, and anti-oxidation effects. Liu et al. found that rhein regulated the proliferation and apoptosis of osteosarcoma cells by inhibiting the expression of STAT3 [11]. Chen et al. found that Rhein inhibits NF-kappaB signaling pathway to alleviate the inflammatory response and oxidative stress of rats with chronic glomerulonephritis [12]. Xu et al. found that Rhein promotes the proliferation of keratinocytes by targeting estrogen receptors for skin ulcer treatment [13]. Therefore, Rhein has great potential for development and clinical application in the field of skin disease treatment. The aim of this study is to investigate the effects of Rhein, the main active component of *R. palmatum* L., on skin wound healing and its therapeutic significance.

Thus, our research aimed to investigate whether Rhein promotes skin wound healing and analyze the potential regulatory mechanism, as to find a novel therapeutic agent for skin wound healing.

## 2 Materials and methods

### 2.1 Animals and wound model of skin

The male C57 mice (6–8 weeks) were provided by the Hubei Beiente Biotechnology Co., Ltd, and housed in constant conditions of temperature, humidity, and light. The skin wound model was established by shaving the back hair of the mice after anesthesia with 1% pentobarbital. Two circular full-thickness skin excisions with a diameter of 5 mm were generated in the middle of the back or on each side of the spine under sterile conditions. All animal handling and experimental protocols were approved by the Animal Care and Use of Laboratory Animals of Bestcell Model Biological Center (approval number: BSMS-2023-10-09D). Then, mice were injected with Rhein (25, 50, and 100 μg/ml) around the wounds for 10 days to conduct Rhein models and untreated mice as normal controls. The wounds in each group were observed and imaged after 0, 4, 7, and 10 days. Wound areas were measured using ImageJ analysis software.

### 2.2 Hematoxylin and eosin (H&E) staining

In brief, the collected skin wound tissues were fixed with 4% paraformaldehyde solution (China National Pharmaceutical Group Chemical Reagent Company, 80096618), dehydrated in a graded ethanol series, and embedded in paraffin to prepare 4 μm sections. Next, the slices were stained with hematoxylin for 3–10 min (Sigma, H9627-25G), differentiated with hydrochloric acid, and stained with eosin staining solution for 1–3 min (Siya reagent, D12621). After that, skin tissue sections were imaged using optical microscopy (OLYMPUS, CX21)

### 2.3 Masson trichrome staining

Collected skin wound tissue was fixed with 4% paraformaldehyde solution (China Pharmaceutical Group Chemical Reagent Company, 80096618), dehydrated in a graded ethanol series, and embedded in paraffin to prepare 4 μm sections. The sections were then preheated and stained with the mixture according to the instruction manual (Servicebio, G1006), followed by 1% glacial acetic acid differentiation for several seconds. After dehydration, the tissue sections were sealed and evaluated for collagen maturation using Masson trichrome staining.

### 2.4 Cell culture and treatment

Human skin fibroblasts (HSFs) were purchased from American type culture collection and maintained in Dulbecco’s modified eagle medium supplemented with 10% fetal bovine serum (Procell) and 1% penicillin/streptomycin (Procell) in an incubator containing 5% CO_2_ at 37°C. Then, cells were treated with different concentrations of Rhein (12.5, 50, 100 μM). After that, the cells were acquired to conduct the following experiment.

### 2.5 Cell counting kit-8 (CCK-8) assay

After treatment, HSFs were inoculated into 96-well plates and cultured for 24 h. Then, each well was added with 10 μl CCK-8 solution and the cells were incubated for 1.5 h. The micro-plate reader (Diatek) was performed to measure the optical density (OD) at 450 nm following the direction to calculate the cell viability.

### 2.6 Wound-healing assay

After treatment, the HSFs were seeded in 96-well plates. Then lines were drawn evenly behind the 96-well plate with a marker. When the cells grow to nearly 90% confluence. The pipette tip was perpendicular to the drawn line to make a linear scratch. Then cells were washed with phosphate-buffered solution (PBS) three times to remove the scratched cells. Then, the serum-free medium was added. Cells were cultured in an incubator containing 5% CO_2_ at 37°C and photographed at 0 and 24 h. The size of the wound gap was measured.

### 2.7 Transwell assay

After treatment, HSFs were incubated in a serum-free medium for starvation and seeded into the upper chamber of transwell chambers with Matrigel. After cultivating for 48 h, the remaining HSFs on the upper chamber were removed with a cotton swab. Then, cells adhering to the under surface of the membrane were fixed with 4% paraformaldehyde and stained with 0.1% crystal violet for 10 min. Cells were observed and counted in five fields under a microscope (Nikon, Japan).

### 2.8 Western blot assay

After treatment, HSFs were washed with ice-cold PBS, treated with RIPA buffer, and centrifuged. Proteins were separated by 10% sodium dodecyl sulfate-polyacrylamide gel electrophoresis and transferred onto polyvinylidene fluoride membranes. After that, the membranes were incubated with 5% fat-free milk for 2 h and then immunoblotted with primary antibodies against p-PI3K, PI3K, p-AKT, AKT, or GAPDH at 4°C overnight. Then, the membranes were washed with TBST and incubated with secondary antibodies for 2 h. The interest proteins were observed using enhanced chemiluminescence. The OD of the protein bands was quantified by ImageJ software.

### 2.9 Statistical analysis

All data are presented as the mean ± SD. Statistical analysis was carried out using SPSS 20.0 (SPSS, Chicago, IL, USA). The differences were analyzed using Student’s *t*-test (two-tailed), and one-way analysis of variance. *p* < 0.05 was considered to indicate a statistically significant difference.


**Ethical approval:** All animal handling and experimental protocols were approved by the Animal Care and Use of Laboratory Animals of Bestcell Model Biological Center (approval number: BSMS-2023-10-09D).

## 3 Results

### 3.1 Rhein accelerated skin wound healing in mice

**Figure 1:**
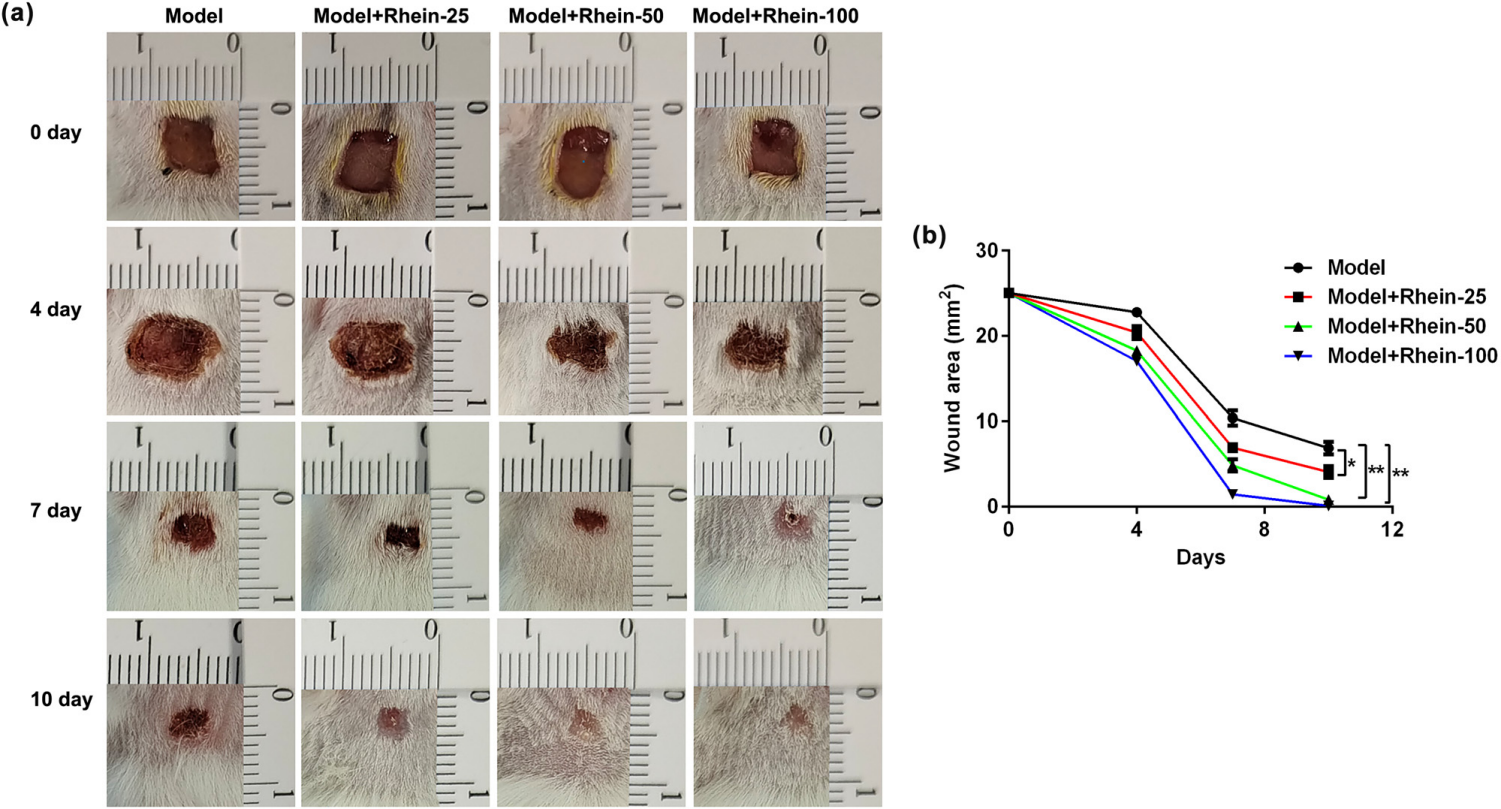
Rhein accelerated cutaneous wound healing in mice. (a) Representative diagram of skin wounds in Rhein-treated mice. (b) Gross view of wound closure in Rhein-treated mice on days 0, 4, 7, and 10 after wounding. *p < 0.05, **p < 0.01.

To investigate the influence of Rhein on skin wound healing, we analyzed wound healing in four groups of mice that were infused with PBS, 25, 50, or 100 μg/ml Rhein around the wound sites. As presented in Figure 1a, Rhein remarkably enhanced wound healing rate in a dose-dependent manner, compared to the model group. Moreover, mice treated with 100 μg/ml Rhein revealed greater wound healing than that in the other three groups at days 4, 7, and 10 post-wounding (Figure 1b; *p* < 0.05). Our data revealed that Rhein promoted skin wound healing in mice.

### 3.2 Rhein promoted epithelialization in mice

**Figure 2:**
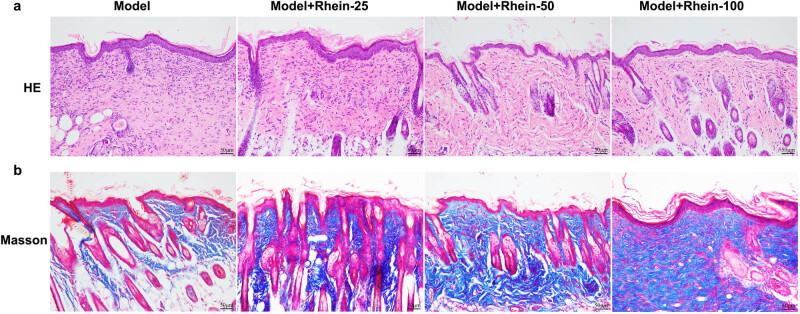
Rhein promoted epithelialization in mice. (a) H&E staining of wound sections. (b) Collagen maturity was assessed by Masson’s trichrome staining.

Moreover, the results of H&E and Masson’s trichrome staining showed that Rhein sensibly promoted re-epithelialization at day 14 post-wounding, as confirmed by the highest wound healing rate, most follicles and largest collagen precipitation areas, as opposed to the Model groups (Figure 2). These results suggested that Rhein has the effect of promoting skin wound healing.

### 3.3 Rhein-activated PI3K/AKT signal pathway in mice skin tissues

**Figure 3:**
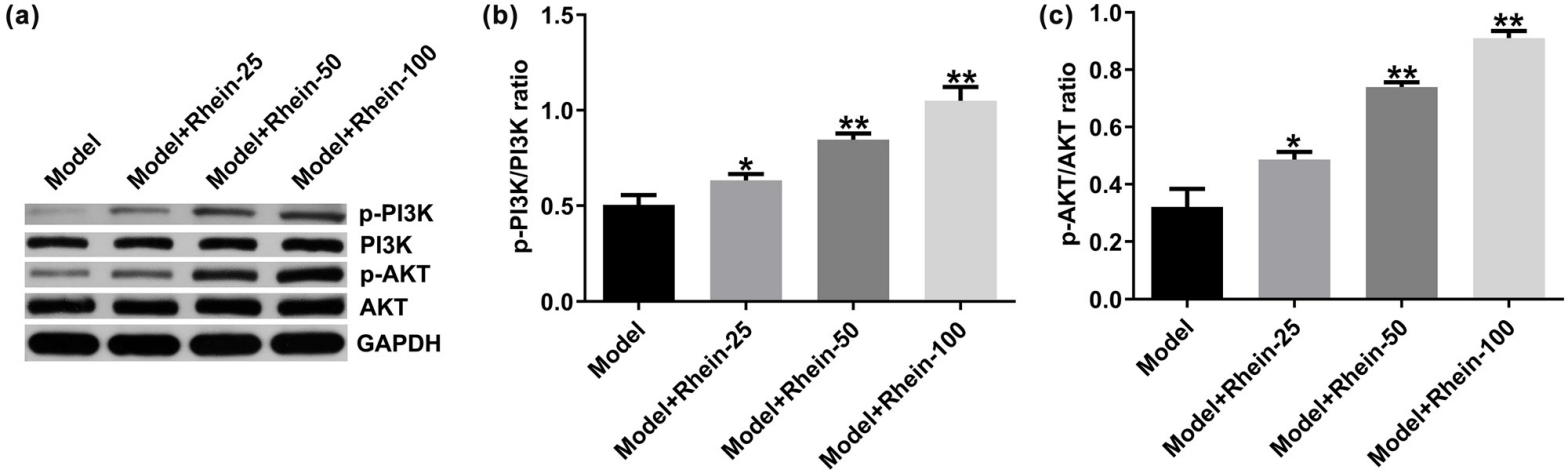
Effects of Rhein on PI3K/AKT signal pathway in cutaneous wound mice. (a) Western blot analysis of p-PI3K and p-AKT expression. (b and c) Quantification of p-PI3K/PI3K and p-AKT/AKT ratio. *p < 0.05, **p < 0.01 vs Model.

To further explain the potential mechanisms of Rhein in promoting skin wound healing, a Western blot assay was conducted to detect p-PI3K, PI3K, p-AKT, and AKT expression in skin wound healing mice after Rhein treatment for 10 days. As displayed in Figure 3a–c, Rhein enhanced p-PI3K and p-AKT expression (Figure 3a), as well as promoted p-PI3K/PI3K (Figure 3b; *p* < 0.05) and p-AKT/AKT ratio (Figure 3c; *p* < 0.01), suggesting that Rhein promoted skin wound healing by activating PI3K/AKT signal pathway.

### 3.4 Rhein promoted HSF proliferation in a dose-dependent manner

**Figure 4:**
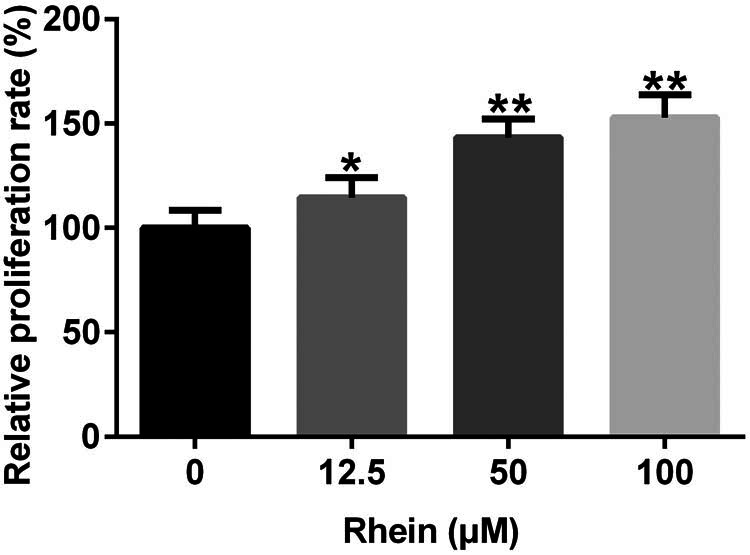
Effects of Rhein on HSF proliferation. HSF cell proliferation was evaluated by CCK-8 assay. *p < 0.05, **p < 0.01 vs control.

To determine the roles of Rhein in skin wound healing *in vitro*, we cultivated HSFs *in vitro* with Rhein at a concentration of 12.5, 50, and 100 μM. Subsequently, HSF cell proliferation was evaluated by CCK-8 assay. As displayed in Figure 4, compared with the control, Rhein promoted HSF cell viability in a dose-dependent manner (*p* < 0.05). Collectively, Rhein regulated HSF cell proliferation.

### 3.5 Rhein promoted HSF migration and invasion in a dose-dependent manner

**Figure 5:**
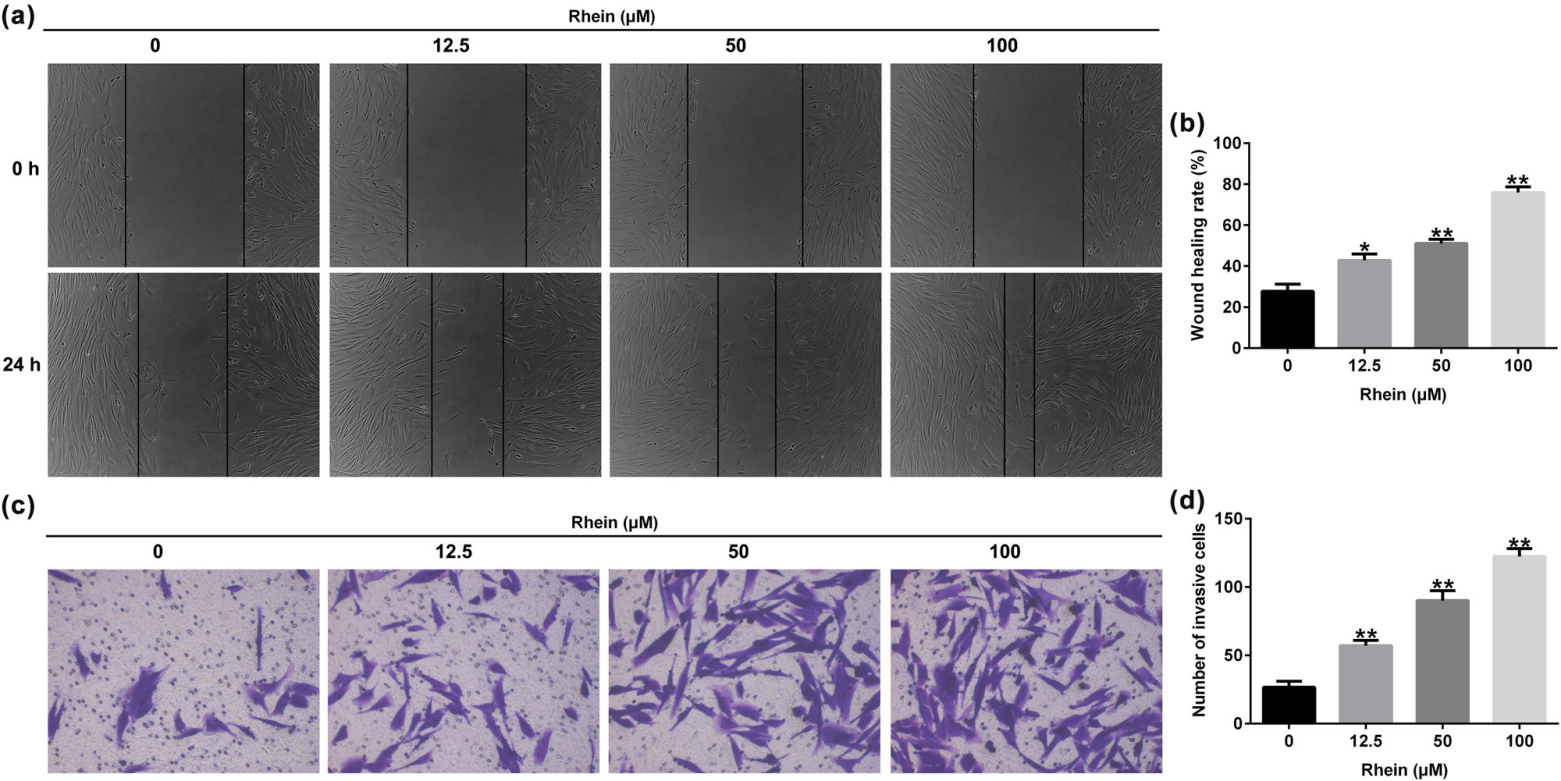
Effects of Rhein on HSF migration and invasion. (a) Scratch assay was conducted to detect HSF cell migration. (b) Quantitative analysis of the migration rate of HSFs. (c) Transwell assay was carried out to determine HSF cell invasion. (d) Number of invasive HSF cells was displayed. *p < 0.05, **p < 0.01 vs control.

In addition, we determined the effects of Rhein on HSF migration and invasion. We cultivated HSFs *in vitro* with Rhein at a concentration of 12.5, 50, and 100 μM, scratch wound and transwell assay were applied to detect HSF migration and invasion. Our data demonstrated that compared with the control, Rhein promoted HSF cell migration and invasion (Figure 5a–d; all *p* < 0.05) in a dose-dependent manner. Our results above demonstrated that Rhein promoted HSF proliferation, migration, and invasion in a dose-dependent manner.

### 3.6 Rhein promoted HSF proliferation, migration, and invasion by activating the PI3K/AKT signal pathway

**Figure 6:**
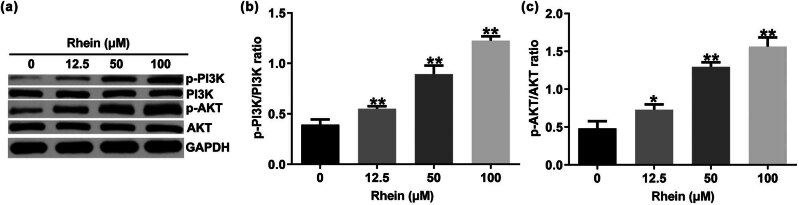
Effects of Rhein on PI3K/AKT signal pathway in HSF cells. (a) Detection of p-PI3K and p-AKT expression using Western blot analysis. (b and c) Quantification of p-PI3K/PI3K and p-AKT/AKT ratio. *p < 0.05, **p < 0.01 vs control.

The PI3K/AKT signaling pathway was reported to play a vital role in cell viability, migration, and invasion [14]. We also determined the protein expression of p-PI3K, PI3K, p-AKT, and AKT in Rhein-treated HSFs. Results from Western blot assay suggested that Rhein had remarkably increased p-PI3K and p-AKT expression (Figure 6a), as well as p-PI3K/PI3K (Figure 6b; *p* < 0.01) and p-AKT/AKT ratio (Figure 6c; *p* < 0.01) in HSF cells, in comparison of the control group (Figure 6a–c), indicating that Rhein regulated HSF cells bio-activity by activating PI3K/AKT signal pathway.

## 4 Discussion

Skin wounds are often caused by various factors, including surgery, disease, or exercise-related injuries. Wound healing is a complex and lengthy process involving cell proliferation and migration, collagen fibers, and epidermal tissue formation [15]. Increasing evidence has demonstrated that skin wound healing requires a well-orchestrated integration of cell migration and proliferation, collagen synthesis and deposition, angiogenesis, and wound remodeling. For instance, Qin et al. revealed that histone deacetylase 6 promotes skin wound healing by regulating fibroblast migration and differentiation in aged mice [16]. However, there is currently limited research on the exact molecular mechanism of skin wounds. Clarifying the molecular mechanisms related to the behavior of human skin fibroblasts is beneficial for discovering new therapeutic strategies for skin wounds.

*R. palmatum* L., a common traditional Chinese medicine, has multiple actions, such as antibacterial, anti-inflammatory, and anti-tumor activities [17]. Rhein is a natural anthraquinone compound, which exists in a variety of medicinal plants and has a variety of pharmacological activities [18]. The pharmacological effects of Rhein and its derivatives have received extensive attention in the field of scientific research. Ma et al. suggested that Rhein inhibits malignant phenotypes of human renal cell carcinoma by impacting MAPK/NF-kappaB signaling pathways [19]. A report from Zhang et al. demonstrated that Rhein inhibits colorectal cancer cell growth by inhibiting the mTOR pathway *in vitro* and *in vivo* [20]. Besides, Xu et al. revealed that Rhein promotes the proliferation of keratinocytes by targeting estrogen receptors for skin ulcer treatment [13]. However, whether Rhein was involved in the progression of the skin wound has not been fully validated. Thus, our research was carried out with the aim of illustrating the latent mechanism of Rhein in the proliferation and migration of HSFs, as well as skin wound mice.

To explore the functions of Rhein in wound healing, we conducted a skin wound model and injected Rhein into the wound skin tissues of mice for 10 days. Then, we assessed wound healing in four groups of mice that were exposed to different concentrations of Rhein. Results from histological assessment of the wound suggested that Rhein remarkably enhanced the wound healing rate in a dose-dependent manner, compared with the control group. Gong et al. suggested that lentivirus-mediated subcutaneous JAM-A modification promotes skin wound healing in a mouse model by strengthening the secretory function and proliferation of fibroblasts [21]. Our results verified that Rhein would promote wound healing with a higher wound closure rate, less fibroblast formation, and more collagen deposition.

Currently, investigations on skin wound-associated signaling pathways mainly focus on the AKT/mTOR pathway, the PI3K/AKT pathway, and the NF-kappaB pathway [22,23]. Xiu et al. have suggested that MSC-derived miR-150-5p-expressing exosomes stimulate skin wound healing by activating the PI3K/AKT pathway [24]. Yu et al. also suggested that insulin promoted macrophage phenotype transition through PI3K/AKT signaling during diabetic wound healing [25]. Thus, we speculated whether Rhein promotes skin wound healing by regulating the PI3K/AKT pathway. The results of the current study revealed that Rhein could activate the PI3K/AKT pathway in both the skin tissue of skin wound mice, demonstrating that Rhein stimulated skin wound healing through activating the PI3K/AKT pathway.

Increasing research has found that cellular bio-activities play a key role in a variety of biological processes, including angiogenesis, inflammation, and skin wound healing. To further explain the mechanism by which Rhein regulates the skin wound, different concentrations of Rhein were cultured with HSF cells, and our data indicated that Rhein remarkably increased the HSF cell proliferation, migration, and invasion. Further functional assay revealed that Rhein remarkably activates the PI3K/AKT pathway in HSF cells.

These *in vivo* and *in vitro* findings demonstrated for the first time that Rhein promoted skin wound healing by activating the PI3K/AKT signaling pathway to stimulate HSF cell proliferation, migration, and invasion, revealing that the application of Rhein may provide new views for therapy to promote skin wound healing.
